# Socially responsible leadership profiles and their correlates among Chinese college students: a latent profile analysis

**DOI:** 10.3389/fpsyg.2026.1950119

**Published:** 2026-10-07

**Authors:** Xing Gao, Xinzhu Wang, Fenglin Li, Bin Gao

**Affiliations:** 1 School of Public Administration, China University of Geosciences, Wuhan, China; 2 School of Teacher Education, Hubei Minzu University, Enshi, China

**Keywords:** college students, extracurricular engagement, latent profile analysis, leadership efficacy, socially responsible leadership, teacher support

## Abstract

**Introduction:**

Socially responsible leadership (SRL) encompasses individual, group, and societal values relevant to college student development. This study identified SRL profiles among Chinese undergraduates, examined profile similarity across gender, and investigated factors associated with profile membership.

**Methods:**

A cross-sectional survey was conducted with 1,248 students from eight higher education institutions. Latent profile analysis was performed using seven SRL dimensions, followed by multigroup comparisons and three-step analyses of profile membership.

**Results:**

Three SRL profiles were identified: low SRL (18.67%), average SRL (57.45%), and high SRL (23.88%). The profiles differed mainly in overall SRL, with broadly parallel patterns across the seven dimensions. Women and men showed the same low–average–high ordering, although corresponding profile means were not fully equivalent. Place of origin, financial hardship, family socioeconomic status, and first-generation status were associated with selected profile contrasts. Leadership efficacy, extracurricular engagement, and perceived teacher support were positively associated with higher-profile membership in separate models.

**Discussion:**

The findings characterize SRL profiles among Chinese college students and reveal both similarities and differences across gender. The associations with extracurricular engagement, leadership efficacy, and teacher support highlight relevant personal and educational resources for student leadership development.

## 1 Introduction

Socially responsible leadership (SRL) conceptualizes leadership as a purposeful, collaborative, and values-based process directed toward positive social change rather than as a formal position or an individual capacity to command others (Higher Education Research Institute, 1996; Komives and Wagner, 2009). This perspective is especially relevant to higher education because it treats leadership as learnable and accessible to students regardless of whether they hold an official title. The Social Change Model organizes SRL around three individual values—consciousness of self, congruence, and commitment—three group values—collaboration, common purpose, and controversy with civility—and the societal value of citizenship. These values are intended to operate as an interdependent system. Self-knowledge and congruence help students act consistently with their principles; commitment sustains purposeful action; collaboration, common purpose, and civil engagement with disagreement enable collective work; and citizenship connects group action to wider community responsibilities. Change is the intended outcome that emerges when these individual, group, and societal values work together (Higher Education Research Institute, 1996; Komives and Wagner, 2009).

College is a relevant setting for studying SRL because students encounter opportunities to develop both individual capacities and relationships for collective action. Leadership identity research describes movement from a position-based understanding toward a relational and collaborative understanding of leadership (Komives et al., 2005). Day's (2000) distinction between leader development and leadership development likewise separates individual human capital from the social capacity required for collective work. College experiences, civic participation, leadership education, and student organizations have been associated with SRL and related outcomes (Cress et al., 2001; Dugan, 2006b; Dugan and Komives, 2010). These findings motivate examination of how the seven values co-occur.

Variable-centered and person-centered approaches address complementary questions. A continuous factor model represents shared variation among SRL dimensions, whereas LPA examines their joint patterns across students (Foti and McCusker, 2017; Howard and Hoffman, 2018; Masyn, 2013; Spurk et al., 2020). Profile differences in elevation indicate broadly higher or lower endorsement across values. Differences in shape indicate relative strengths and weaknesses across dimensions, such as strong individual commitment alongside weaker collaboration or citizenship. Examining both features helps clarify whether variation in SRL primarily concerns its overall strength or the balance among its individual, group, and societal values. Person-centered leadership studies have identified patterns of leadership behaviors, executive personality, school leadership, and safety leadership that differ in level, balance, or both (Doucet et al., 2015; Parr et al., 2016; Urick and Bowers, 2014; Willis et al., 2021; Howard et al., 2025). Extending this approach to college students allows the number and characteristics of SRL profiles to be explored across the seven values.

Chinese higher education provides a relevant context for examining SRL. National policy on practice-based education connects higher education with social responsibility and participation in practical and social activities (Ministry of Education of the People's Republic of China, 2012). These organized experiences provide opportunities to develop commitment, collective responsibility, and citizenship. Cultural and institutional settings may also shape how students understand and practice these values, as suggested by cross-national SRL research (Dugan et al., 2011).

Within this context, extracurricular engagement and teacher support may help explain differences in students’ leadership orientations. The participating institutions varied in geographic location, educational orientation, and public or private status, allowing these associations to be examined across different higher education settings.

Gender is relevant to SRL because leadership experiences and social expectations may differ for women and men. Previous studies often report higher scores among women on relational and socially responsible values, although findings vary across dimensions and experiences (Dugan, 2006a; Dugan et al., 2008; Haber and Komives, 2009; Shim, 2013). Social-role and role-congruity perspectives connect these patterns to expectations for communal and agentic behavior (Ayman and Korabik, 2010; Eagly and Karau, 2002), while student leadership research emphasizes experience and context (Kezar and Moriarty, 2000). This evidence motivates H1. Comparing profile membership across gender also requires examining whether corresponding profiles have similar meanings and indicator levels (Morin et al., 2016), which is addressed in RQ2.

Other demographic factors may influence SRL through unequal access to developmental resources, although the available evidence is less consistent and does not justify uniformly directional hypotheses. Year level may represent accumulated exposure to curricula, peer networks, organizations, service, and leadership opportunities. Longitudinal evidence shows that leadership attitudes and beliefs can change between entry into college and later years (Fischer et al., 2015), but class standing alone does not reveal the quality of students’ experiences. University type and academic major may similarly structure the availability and perceived relevance of leadership activities. Socioeconomic resources, financial hardship, rural or urban origin, and first-generation status may affect discretionary time, institutional knowledge, access to mentoring and networks, and the feasibility of unpaid extracurricular participation. First-generation students, for example, often encounter differences in participation and college experiences associated with financial demands and mismatches between home-based and institutional expectations (Pascarella et al., 2004; Stephens et al., 2012). At the same time, these characteristics should not be treated as deficits or assumed to determine leadership capacity; students may also draw on family, work, and community experiences that conventional campus measures overlook. Because little research has tested whether university type, major, ethnicity, place of origin, financial hardship, family socioeconomic status, and first-generation status differentiate membership in the estimated SRL profiles, the present study examined these variables exploratorily while advancing a directional hypothesis only for year level.

Extracurricular engagement provides an experiential link to SRL. Astin's (1984) involvement theory emphasizes the energy students invest in educational activities rather than attendance alone. Student organizations, service activities, and shared projects can create occasions to practice responsibility, coordination, and disagreement, consistent with earlier associations between involvement and leadership outcomes (Cress et al., 2001; Dugan, 2006b; Dugan and Komives, 2010; Haber and Komives, 2009; Pless et al., 2011). Cognitive, emotional, and behavioral engagement may help students reflect on values, sustain commitment, and contribute to collective tasks (Reeve and Tseng, 2011). These links motivate H3: greater engagement should be associated with membership in higher-SRL profiles.

Leadership efficacy supplies a motivational link. It concerns confidence in performing leadership tasks, whereas SRL concerns endorsement and enactment of socially responsible values. Social cognitive theory proposes that efficacy beliefs influence goal selection, effort, and persistence, and that mastery experiences and feedback can strengthen efficacy (Bandura, 1997; McCormick, 2001; Hannah et al., 2008; Machida and Schaubroeck, 2011). Student leadership research also links experience with efficacy trajectories (Rosch et al., 2017). Confidence may thus accompany greater willingness to contribute, collaborate, and persist, motivating H4.

Perceived teacher support represents a relational resource that may complement students’ behavioral engagement and efficacy beliefs. Faculty members can affirm students’ capacities, provide feedback and guidance, connect students with developmental opportunities, and model collaborative and ethical practice. Research on college student leadership identifies mentoring and student–faculty relationships as meaningful influences on leadership identity and socially responsible leadership capacity (Komives et al., 2005; Dugan and Komives, 2010). More broadly, supportive teacher–student relationships and student–faculty interactions contribute to engagement, belonging, integration, and other developmental outcomes in higher education (Hagenauer and Volet, 2014; Kahu and Nelson, 2018; Kim and Sax, 2009). Within the Social Change Model, such support may strengthen self-awareness and commitment while creating opportunities to practice collaboration, common purpose, and civic engagement. Perceived teacher support was therefore expected to distinguish students in higher- and lower-SRL profiles.

The Social Change Model, involvement theory, social cognitive theory, and the relational perspective together connect leadershi*p* values with the experiences and resources through which they may develop. The Social Change Model specifies the individual, group, and societal values underlying SRL. Engagement provides opportunities to practice these values, efficacy can sustain effort and persistence, and supportive teacher relationships offer feedback and access to further opportunities (Komives et al., 2005; Dugan and Komives, 2010). These processes may reinforce one another: support can facilitate engagement, participation can provide mastery experiences, and growing confidence can encourage further involvement. Shared developmental opportunities may be associated with broadly elevated SRL, whereas uneven experiences may be associated with different patterns of strengths across values.

Guided by this framework, the study examines SRL profiles (RQ1), their similarity across gender (RQ2), and demographic correlates of profile membership (RQ3). The hypotheses address gender, year level, extracurricular engagement, leadership efficacy, and perceived teacher support as potential correlates of membership in higher-SRL profiles.

*RQ1*: What SRL profiles emerge among college students, and do they differ primarily in overall elevation, in shape across the seven dimensions, or in both?

*RQ2*: Are the number and characteristics of the identified SRL profiles comparable across women and men?

*H1*: Women will be more likely than men to belong to profiles characterized by higher overall levels of SRL.

*H2*: Students in later years of study will be more likely to belong to profiles characterized by higher overall levels of SRL.

*RQ3*: How are university type, major, ethnicity, place of origin, financial hardship, family socioeconomic status, and first-generation college student status related to SRL profile membership?

*H3*: Students reporting greater extracurricular engagement will be more likely to belong to profiles characterized by higher overall levels of SRL.

*H4*: Students reporting higher leadership efficacy will be more likely to belong to profiles characterized by higher overall levels of SRL.

*H5*: Students reporting stronger perceived teacher support will be more likely to belong to profiles characterized by higher overall levels of SRL.

## 2 Materials and methods

### 2.1 Participants and procedure

The initial sample included 1,352 undergraduate students. After data screening, 104 cases were excluded because their questionnaires were completed in less than 5 min, contained incomplete information, or exhibited invariant response patterns, such as selecting the same response option throughout the questionnaire. The five-minute criterion was based on the expected time needed to read and answer the questionnaire at a normal pace; shorter completion times were considered a risk indicator for inattentive responding. Response time was considered alongside other indicators of response quality because different indicators can detect different patterns of careless responding (Meade and Craig, 2012). Attention-check items were also included in the questionnaire to provide complementary information for response-quality screening. The final analytic sample comprised 1,248 students, corresponding to a retention proportion of 92.3%. Of the participants, 675 were women (54.1%) and 573 were men (45.9%). The sample included 321 first-year students (25.7%), 421 s-year students (33.7%), 333 third-year students (26.7%), and 173 fourth-year students (13.9%). Regarding place of origin, 723 students were from urban areas (57.9%) and 525 were from rural areas (42.1%). In terms of academic major, 589 students studied science, technology, engineering, and mathematics disciplines (47.2%), whereas 659 studied humanities and social sciences (52.8%). In addition, 819 participants were first-generation college students (65.6%), and 429 were non-first-generation college students (34.4%).

This study employed a cross-sectional survey design. Using convenience sampling, participants were recruited from eight higher education institutions in eastern, central, and western China between December 2024 and June 2025. The institutions included research-oriented and teaching-oriented universities, as well as public and private institutions. Selection sought to include these different regional and institutional contexts within the research team’s accessible recruitment network. Each institution contributed between 100 and 200 students to the analytic sample. Members of the research team distributed an electronic questionnaire through Wenjuanxing, an online survey platform in China. Participants were informed of the study purpose, the voluntary and anonymous nature of participation, and their right to withdraw. Electronic informed consent was obtained before participation. The study protocol was approved by the Ethics Committee of Shanghai Normal University (approval no. 2023038).

### 2.2 Measures

#### 2.2.1 Demographic characteristics

The demographic variables were university type (Double First-Class vs. other), gender, grade, major (humanities/social sciences vs. STEM), ethnicity (minority vs. Han), place of origin (rural vs. urban), financial hardship, family socioeconomic status (SES), and first-generation college status.

#### 2.2.2 Socially responsible leadership

SRL was assessed with a culturally adapted version of the Socially Responsible Leadership Scale used by Anderson (2012). The 48 items assessed consciousness of self, congruence, commitment, collaboration, common purpose, controversy with civility, and citizenship on a 5-point scale (1 = strongly disagree, 5 = strongly agree). A seven-factor confirmatory model fit the data adequately (CFI = 0.93, TLI = 0.93, SRMR = 0.030, RMSEA = 0.043). Cronbach’s α values for the seven dimensions ranged from 0.88 to 0.96.

#### 2.2.3 Leadership efficacy

Leadership efficacy was measured with four items adapted from Wilson (2009), assessing confidence in leading others, organizing a team to achieve goals, initiating improvement, and collaborating to complete collective tasks. Items were rated on a 5-point scale (1 = strongly disagree, 5 = strongly agree). The scale demonstrated excellent internal consistency (Cronbach’s *α* = 0.94).

#### 2.2.4 Extracurricular engagement

Extracurricular engagement was assessed using 12 items adapted from Reeve and Tseng’s (2011) student engagement measure to reflect students’ involvement in extracurricular activities. The adapted measure comprised three dimensions: cognitive engagement (five items), emotional engagement (three items), and behavioral engagement (four items). Responses were recorded on a 5-point Likert-type scale, with higher composite scores indicating greater engagement in extracurricular activities. Confirmatory factor analysis supported the proposed three-factor structure, with acceptable model fit: CFI = 0.94, TLI = 0.93, RMSEA = 0.078, and SRMR = 0.005. The overall measure demonstrated excellent internal consistency in the present sample (Cronbach’s *α* = 0.97).

#### 2.2.5 Perceived teacher support

Perceived teacher support was assessed using six items adapted from the Brief Form of the Perceived Social Support Questionnaire (F-SozU K-6; Lin et al., 2019). The items were reworded to reflect students’ perceptions of teachers’ understanding, care, and emotional or practical support. Participants responded on a 5-point Likert scale ranging from 1 (not at all true) to 5 (completely true), with higher mean scores indicating stronger perceived teacher support. Confirmatory factor analysis showed satisfactory model fit (CFI = 0.96, TLI = 0.95, RMSEA = 0.036, SRMR = 0.045), and the scale demonstrated excellent internal consistency (Cronbach’s *α* = 0.92).

### 2.3 Data analysis

The primary analyses were conducted in Mplus 8.3 (Muthén and Muthén, 1998–2017) using robust maximum likelihood (MLR). Gender measurement invariance was examined through configural, metric, scalar, and residual models. Model comparisons considered scaled chi-square difference tests and changes in CFI, RMSEA, and SRMR (Chen, 2007; Cheung and Rensvold, 2002). Common method bias was assessed by comparing a single-factor CFA model with a correlated four-factor model representing SRL, leadership efficacy, extracurricular engagement, and perceived teacher support. Both models were estimated in lavaan using MLR on the same complete-case sample, with demographic variables included as covariates. Fit was evaluated using robust CFI, TLI, and RMSEA, together with SRMR. The residual association between SRL and efficacy was examined within the covariate-adjusted four-factor model.

LPA was conducted using standardized scores for the seven SRL dimensions. One- through five-profile models were estimated with 4,000 initial and 1,000 final random starts, equal within-profile variances, and zero residual covariances. Model selection considered AIC, BIC, sample-size-adjusted BIC (aBIC), the adjusted Lo–Mendell–Rubin likelihood-ratio test (LMR–LRT), the bootstrap likelihood-ratio test (BLRT), entropy, profile size, convergence, and interpretability (Masyn, 2013; Nylund et al., 2007; Nylund-Gibson and Choi, 2018). No single test was treated as decisive when these criteria disagreed. Multigroup LPA examined gender similarity in profile number and pattern (configural similarity), corresponding means (structural similarity), within-profile variances (dispersion similarity), and proportions (distributional similarity; Morin et al., 2016). Equality constraints were added sequentially.

Associations with profile membership were estimated using a three-step approach accounting for classification uncertainty (Asparouhov and Muthén, 2014). Demographic variables were entered jointly. Standardized leadership efficacy, extracurricular engagement, and perceived teacher support were each examined in a separate unadjusted model. Two-tailed p values below 0.05 were considered statistically significant.

## 3 Results

### 3.1 Measurement model comparisons and invariance across gender

The single-factor model showed poor fit (CFI = 0.513, TLI = 0.500, RMSEA = 0.142, SRMR = 0.167), whereas the four-factor model fit substantially better (CFI = 0.914, TLI = 0.910, RMSEA = 0.060, SRMR = 0.026). These results supported a multidimensional structure and indicated that a single common factor did not adequately account for the item covariance. In the four-factor model, the residual correlation between SRL and leadership efficacy after adjustment for demographic covariates was 0.805 (95% CI [0.767, 0.844]). The gender measurement-invariance models showed small changes in approximate fit (Table 1). Changes in CFI and RMSEA were both −0.003 for the metric and scalar comparisons. The residual-invariance model yielded CFI = 0.974, TLI = 0.977, RMSEA = 0.070, and SRMR = 0.025. Although the metric-versus-configural chi-square difference was significant (*p* = 0.029), the approximate fit indices indicated limited deterioration across the tested constraints.

**Table 1:** Fit indices and model comparisons for gender measurement invariance.

Model	Model fit					Comparison with the preceding model				
	*χ*^2^ (df)	CFI	TLI	RMSEA	SRMR	Comparison	Δ*χ*^2^ MLR (Δdf)	*p*	ΔCFI	ΔRMSEA
Configural invariance	162.39 (28)	0.976	0.965	0.088	0.014	–	–	–	–	–
Metric invariance	187.78 (34)	0.973	0.967	0.085	0.022	Metric vs. configural	14.06 (6)	0.029	−0.003	−0.003
Scalar invariance	208.49 (40)	0.970	0.969	0.082	0.023	Scalar vs. metric	10.48 (6)	0.106	−0.003	−0.003
Residual (strict) invariance	192.6 (47)	0.974	0.977	0.070	0.025	Strict vs. scalar	8.07 (7)	0.326	0.004	−0.012

Models were estimated using robust maximum likelihood (MLR). Δ*χ*^2^ MLR denotes the robust scaled chi-square difference test. CFI, comparative fit index; TLI, Tucker–Lewis index; RMSEA, root mean square error of approximation; SRMR, standardized root mean square residual.

### 3.2 Latent profile analysis

One- through five-profile solutions were estimated (Table 2). Information criteria decreased as profiles were added. The LMR-LRT favored two profiles over one but was nonsignificant for three versus two (*p* = 0.172), whereas the BLRT supported three over two profiles. The nonsignificant LMR-LRT was considered alongside the lower information criteria and the substantive interpretability of the three-profile solution. Within this combined assessment, greater weight was given to the BLRT because simulation evidence indicates consistent performance in identifying the number of classes across the mixture-model conditions examined (Nylund et al., 2007). The three-profile solution distinguished low, average, and high SRL patterns, with each profile containing at least 18.67% of the sample. Although information criteria continued to improve for four and five profiles, these solutions isolated an eight-person profile (0.64%) with extreme means and numerical instability. Considering fit, profile size, convergence, and interpretability, the three-profile solution was retained, with entropy of 0.984. The nonsignificant three-versus-two LMR-LRT nevertheless indicated that support for three profiles was not uniform across selection criteria.

**Table 2:** Fit indices for the latent profile models.

Classes	AIC	BIC	aBIC	Entropy	LMR-LRT	BLRT	Smallest class %
1	24,812.69	24,884.50	24,840.03	–	–	–	100.00
2	19,842.78	19,955.62	19,885.74	0.975	<0.001	<0.001	20.91
3	13,801.62	13,955.50	13,860.20	0.984	0.172	<0.001	18.67
4	11,006.28	11,201.19	11,080.49	0.992	<0.001	<0.001	0.64
5	9,669.75	9,905.70	9,759.58	0.987	<0.001	<0.001	0.64

LMR-LRT, adjusted Lo–Mendell–Rubin likelihood-ratio test; BLRT, bootstrap likelihood-ratio test. The smallest class percentage is based on most likely class membership.

The average SRL profile was the largest group (57.45%), with standardized means near zero (−0.07 to −0.01). The low SRL profile comprised 18.67% of students, with means from −1.56 to −1.29, and the high SRL profile comprised 23.88%, with means from 1.13 to 1.30. The profiles followed broadly parallel patterns across the seven values and differed mainly in overall elevation (Figure 1). Profile labels describe relative standing within the sample.

**Figure 1:**
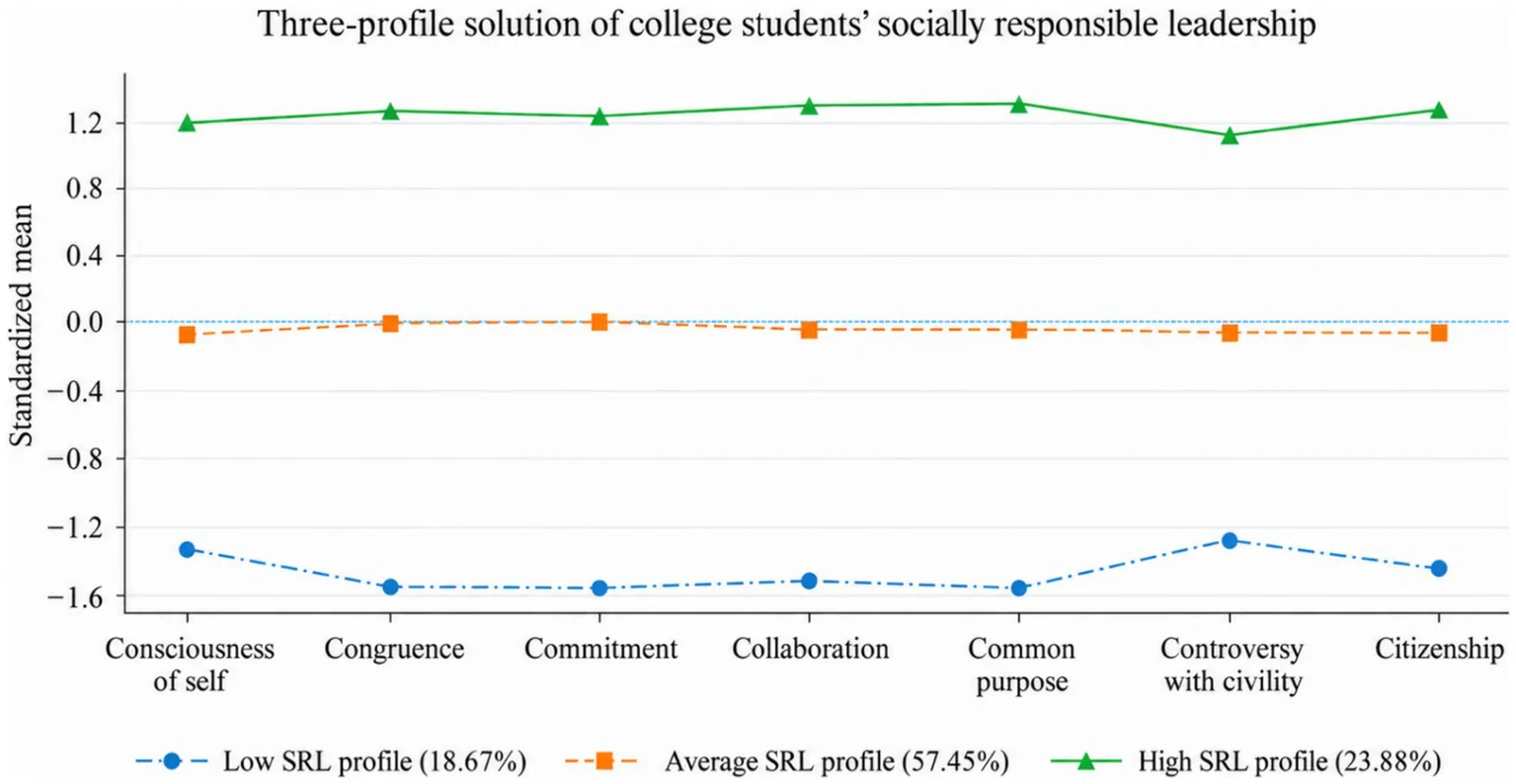
Standardized SRL dimension means in the retained three-profile solution. SRL, socially responsible leadership.

### 3.3 Gender comparison of the three-profile SRL solution

The three-profile solution showed a similar low–average–high ordering among women and men (Table 3). In the configural model, the estimated proportions were 15.9, 61.2, and 22.9% among women and 23.3, 50.9, and 25.8% among men. These proportions describe the gender-specific solutions, in which corresponding profile means and variances were freely estimated. Full structural similarity was not supported: constraining corresponding profile means to equality significantly worsened fit, ΔTRd(21) = 122.45, *p* < 0.001. The dispersion and distributional models also showed significant fit losses (both *p* < 0.001), conditional on the preceding mean-equality constraints. Thus, the common profile ordering did not imply equivalent profile definitions across gender, and H1 was not supported as a comparison of membership in equivalent profiles.

**Table 3:** Gender comparisons for the three-profile SRL solution.

Model	FP	LL	AIC	BIC	aBIC	Entropy	Comparison	ΔTRd (df)	*p*
M1: Configural	61	−7,437.67	14,997.35	15,310.23	15,116.47	0.991	–	–	–
M2: Structural	40	−7,508.32	15,096.64	15,301.81	15,174.75	0.991	M2 vs. M1	122.45 (21)	<0.001
M3: Dispersion	33	−7,708.29	15,482.59	15,651.86	15,547.04	0.991	M3 vs. M2	75.60 (7)	<0.001
M4: Distributional	31	−7,717.07	15,496.14	15,655.15	15,556.68	0.990	M4 vs. M3	18.30 (2)	<0.001

SRL, socially responsible leadership; FP, number of free parameters; LL, loglikelihood; aBIC, sample-size-adjusted Bayesian information criterion. ΔTRd denotes the MLR-scaled likelihood-ratio difference test against the immediately preceding model. Lower information criteria indicate better relative fit. The dispersion and distributional models retain the full structural constraints; their comparisons are conditional on those constraints.

### 3.4 Factors associated with leadership profile membership

The demographic variables were examined jointly, whereas leadership efficacy, extracurricular engagement, and perceived teacher support were examined in separate models (Table 4). Rural origin was associated with lower odds of high-profile membership relative to the low and average profiles. Financial hardship was associated with higher odds of high-profile membership. Higher family SES and non-first-generation status were associated with membership in the average and high profiles relative to the low profile. University type, grade, major, and ethnicity were not significantly associated with the reported profile contrasts. In the pooled-sample model, men had lower odds of average- versus low-profile membership and higher odds of high- versus average-profile membership. These coefficients concern the pooled profiles rather than equivalent gender-specific profiles. Higher leadership efficacy, extracurricular engagement, and perceived teacher support were each associated with higher-profile membership across all three contrasts in their separate models. For the high-versus-low contrast, the odds ratios were 194.88 for efficacy (95% CI [100.04, 379.64]), 3.52 for engagement (95% CI [2.82, 4.38]), and 3.14 for teacher support (95% CI [2.56, 3.85]). The directions of these associations were consistent with H3–H5.

**Table 4:** Factors associated with leadership profile membership.

Variable	Average vs. Low				High vs. Low				High vs. Average			
	*B*	SE	OR	95% CI	*B*	SE	OR	95% CI	*B*	SE	OR	95% CI
Double first-class university (yes vs. no)	−0.20	0.20	0.82	[0.56, 1.21]	−0.31	0.24	0.74	[0.46, 1.18]	−0.11	0.20	0.90	[0.61, 1.32]
Sex (male vs. female)	−0.66^***^	0.16	**0.52**	[0.38, 0.71]	−0.28	0.18	0.75	[0.52, 1.08]	0.37^*^	0.15	**1.45**	[1.09, 1.94]
Grade	−0.10	0.09	0.90	[0.76, 1.07]	−0.10	0.10	0.90	[0.74, 1.10]	0.00	0.08	1.00	[0.86, 1.17]
Major (humanities/social sciences vs. STEM)	0.00	0.17	1.00	[0.72, 1.39]	0.13	0.19	1.14	[0.78, 1.67]	0.13	0.15	1.14	[0.85, 1.52]
Ethnicity (minority vs. Han)	0.11	0.26	1.11	[0.67, 1.85]	0.29	0.32	1.33	[0.71, 2.48]	0.18	0.27	1.19	[0.70, 2.02]
Place of origin (rural vs. urban)	0.30	0.19	1.35	[0.92, 1.97]	−0.51^*^	0.25	**0.60**	[0.37, 0.98]	−0.81^***^	0.21	**0.45**	[0.29, 0.67]
Financial hardship (yes vs. no)	0.17	0.26	1.19	[0.72, 1.98]	0.86^**^	0.33	**2.35**	[1.23, 4.49]	0.68^*^	0.27	**1.98**	[1.17, 3.35]
Family SES	0.29^***^	0.08	**1.34**	[1.15, 1.56]	0.34^***^	0.09	**1.40**	[1.17, 1.69]	0.05	0.07	1.05	[0.91, 1.21]
First-generation status (non-first-generation vs. first-generation)	0.52^*^	0.21	**1.69**	[1.12, 2.54]	0.95^***^	0.23	**2.58**	[1.65, 4.03]	0.42^**^	0.16	**1.53**	[1.12, 2.10]
Leadership efficacy	2.41^***^	0.25	**11.14**	[6.77, 18.34]	5.27^***^	0.34	**194.88**	[100.04, 379.64]	2.86^***^	0.20	**17.49**	[11.80, 25.92]
Extracurricular engagement	0.77^***^	0.07	**2.17**	[1.90, 2.47]	1.26^***^	0.11	**3.52**	[2.82, 4.38]	0.48^***^	0.10	**1.62**	[1.32, 1.99]
Perceived teacher support	0.70***	0.07	**2.01**	[1.75, 2.32]	1.14***	0.10	**3.14**	[2.56, 3.85]	0.44***	0.09	**1.56**	[1.30, 1.87]

Average profile = Class 1; low profile = Class 2; high profile = Class 3. B, logit coefficient; SE, standard error; OR, odds ratio; CI, confidence interval; SES, socioeconomic status; STEM, science, technology, engineering, and mathematics. *p* values are based on logit-coefficient tests. Demographics were entered jointly; efficacy, engagement, and teacher support were each examined in a separate model. Their ORs refer to a one-unit increase in the standardized score. Bold ORs indicate *p* < 0.05. Gender coefficients concern pooled-sample profiles. *p < 0.05; **p < 0.01; ***p < 0.001.

## 4 Discussion

The study identified three SRL profiles among Chinese college students: low, average, and high. Women and men shared a similar ordering, although corresponding profile means differed. Profile membership was associated with place of origin, financial hardship, family SES, and first-generation status, but not year level. Leadership efficacy, extracurricular engagement, and perceived teacher support were positively associated with membership in higher-SRL profiles.

Higher endorsement of individual SRL values generally accompanied higher endorsement of group and societal values. This coordinated pattern is consistent with the interrelated content of the Social Change Model. The broadly parallel profiles indicate that students differed primarily in the strength of their socially responsible leadership orientations rather than in qualitatively different combinations of values.

LPA describes how the seven SRL values occur together across students and estimates the proportions associated with the low, average, and high patterns. This offers a person-centered summary of variation in SRL. A continuous factor or total score captures overall differences while retaining variation within each profile. The two approaches are therefore complementary: the present profiles characterize groups with similar score patterns, but their parallel shapes do not establish greater explanatory value than a continuous SRL model.

The gender findings suggest a shared broad organization of SRL alongside differences in corresponding profile means. Consequently, differences in group proportions cannot be read as a gender advantage in otherwise identical profiles. In particular, the pooled-sample gender coefficients in Table 4 cannot be interpreted as gender differences in equivalent SRL profiles, because full structural similarity of the gender-specific profiles was not supported. Partial structural-similarity models could clarify which SRL dimensions account for these differences by allowing selected means to vary across gender (Morin et al., 2016). Comparisons of profile proportions could then be based on the resulting model. Higher SES and non-first-generation status were associated with higher SRL classifications, whereas rural origin was associated with lower odds of high-profile membership. These patterns may reflect differences in access to time, networks, and developmental opportunities, consistent with research on college experiences and institutional expectations (Pascarella et al., 2004; Stephens et al., 2012). Moreover, the positive association with financial hardship was conditional on the other demographic variables, suggesting a more complex relationship between economic circumstances and SRL than a simple resource gradient. Year level was not associated with profile membership; the content and quality of students’ experiences may be more relevant than time in college alone.

The positive engagement association is consistent with involvement theory: sustained participation creates opportunities to practice shared responsibility, collaboration, and civic contribution (Astin, 1984; Haber and Komives, 2009). Students with stronger SRL orientations may also be more inclined to engage in these activities, suggesting a potentially reciprocal relationship. Leadership efficacy showed a particularly strong association with profile membership. Confidence in organizing, initiating, and collaborating is closely related to several SRL dimensions, although efficacy concerns perceived capability and SRL emphasizes socially responsible values. The strong residual SRL–efficacy correlation in the demographic-adjusted measurement model also indicated substantial shared variance. Conceptual proximity and same-source, same-occasion self-reports may both contribute to the large association (Podsakoff et al., 2003). The efficacy estimate is therefore best understood as an association between closely related self-perceptions rather than an independent causal effect. Shared method variance and conceptual overlap are plausible sources of upward bias in the magnitude of this association; the present design does not distinguish their contributions from a substantive relationship between the constructs. Perceived teacher support was positively associated with higher-profile membership, consistent with relational accounts of leadership development (Komives et al., 2005; Dugan and Komives, 2010). Encouragement, feedback, and access to activities may support students’ willingness to contribute and collaborate. This interpretation accords with research linking teacher–student relationships to engagement and development in higher education (Hagenauer and Volet, 2014; Kahu and Nelson, 2018; Kim and Sax, 2009).

### 4.1 Limitations and future directions

First, participants were recruited through convenience sampling from eight higher education institutions. Although these institutions covered eastern, central, and western China and included research-oriented and teaching-oriented universities, as well as public and private institutions, this variation does not establish representativeness. Students’ willingness to participate and the recruitment networks available to the research team may have influenced the composition of the sample. Future studies could use stratified sampling across regions and institution types to examine the generalizability of the profile structure and its associated factors. In addition, the five-minute screening criterion reflected the expected reading and completion time rather than an independently validated threshold. Although attention-check items provided an additional indicator of response quality, reading speed and response styles vary across individuals. Future research could examine the sensitivity of the findings to alternative completion-time criteria in conjunction with other response-quality indicators. Second, the cross-sectional design limits conclusions about the direction of the observed associations. Leadership efficacy, extracurricular engagement, and teacher support may contribute to students’ SRL, but students with stronger leadership orientations may also seek more opportunities for participation, develop greater confidence, and establish more supportive relationships with teachers. These relationships may therefore be reciprocal. Moreover, all focal constructs were assessed through self-report at the same measurement occasion. The strong association between efficacy and SRL may partly reflect their conceptual proximity and shared self-evaluative tendencies. Longitudinal studies incorporating peer or teacher assessments and behavioral indicators could clarify how leadership confidence relates to responsible leadership practice over time. Because efficacy, engagement, and teacher support were examined in separate models, their associations should also be distinguished from mutually adjusted contributions. Future analyses could examine these resources jointly to clarify their shared and distinct relationships with SRL. Third, the retained profiles were broadly parallel across the seven SRL dimensions and primarily reflected differences in overall elevation. The low, average, and high profiles offer a useful summary of students’ reported leadership orientations, but they should not be interpreted as qualitatively distinct leadership types or successive developmental stages. Further research could compare profile-based and continuous representations of SRL in relation to external outcomes, such as collaborative behavior, civic participation, and sustained leadership involvement. Such comparisons would help establish whether profile membership provides information beyond an overall SRL score. Repeated assessments could also examine the stability of the profiles and whether changes in students’ experiences are accompanied by changes in profile membership. Finally, women and men showed a similar broad profile ordering, but full structural similarity was not supported. Corresponding profile labels therefore did not necessarily represent equivalent SRL means across gender, limiting direct comparisons of profile proportions. Future research could examine partial structural similarity by allowing selected profile means to differ while retaining supported equality constraints on others. This approach could identify the dimensions underlying gender differences and provide a stronger basis for subsequent comparisons of profile variability and prevalence. Examining students’ access to leadership roles, extracurricular experiences, and mentoring alongside these comparisons could further illuminate the educational contexts associated with gender-related patterns in SRL.

## 5 Conclusion

This study identified three socially responsible leadership (SRL) profiles among Chinese college students: low SRL (18.67%), average SRL (57.45%), and high SRL (23.88%). The profiles displayed broadly parallel patterns across the seven dimensions, suggesting that differences among students primarily concerned the overall strength of their leadership orientations. Higher endorsement of individual values generally accompanied stronger group-oriented and societal values, consistent with the interconnected nature of the Social Change Model. Women and men shared the same broad profile ordering, although differences in corresponding profile means indicated that this similarity did not extend to full structural equivalence.

Profile membership was associated with both students’ backgrounds and their personal and educational resources. Place of origin, financial hardship, family socioeconomic status, and first-generation status were associated with selected profile contrasts. Leadership efficacy, extracurricular engagement, and perceived teacher support were positively associated with higher-SRL profile membership in separate models. Together, these findings situate students’ leadership orientations within their broader educational experiences and social circumstances. The findings suggest that leadership education should connect reflection on personal values with opportunities to collaborate, manage disagreement constructively, and contribute to communities. Universities could embed these experiences in existing student organizations and service activities, supported by faculty guidance, feedback, and progressively challenging responsibilities. Providing flexible and accessible participation opportunities may help students with different backgrounds and levels of experience engage in responsible leadership practice. Longitudinal research can further examine how these experiences relate to changes in SRL and whether the observed profiles remain consistent over time.

## Data Availability

The raw data supporting the conclusions of this article will be made available by the authors, without undue reservation.
